# Anti-tumor Effect of Oleic Acid in Hepatocellular Carcinoma Cell Lines *via* Autophagy Reduction

**DOI:** 10.3389/fcell.2021.629182

**Published:** 2021-02-05

**Authors:** Federico Giulitti, Simonetta Petrungaro, Sara Mandatori, Luana Tomaipitinca, Valerio de Franchis, Antonella D'Amore, Antonio Filippini, Eugenio Gaudio, Elio Ziparo, Claudia Giampietri

**Affiliations:** Department of Anatomical, Histological, Forensic Medicine, and Orthopedic Sciences, Sapienza University of Rome, Rome, Italy

**Keywords:** lipid droplets, autophagy, fatty acids, cell death, cancer

## Abstract

Oleic acid (OA) is a component of the olive oil. Beneficial health effects of olive oil are well-known, such as protection against liver steatosis and against some cancer types. In the present study, we focused on OA effects in hepatocellular carcinoma (HCC), investigating responses to OA treatment (50–300 μM) in HCC cell lines (Hep3B and Huh7.5) and in a healthy liver-derived human cell line (THLE-2). Upon OA administration higher lipid accumulation, perilipin-2 increase, and autophagy reduction were observed in HCC cells as compared to healthy cells. OA in the presence of 10% FBS significantly reduced viability of HCC cell lines at 300 μM through Alamar Blue staining evaluation, and reduced cyclin D1 expression in a dose-dependent manner while it was ineffective on healthy hepatocytes. Furthermore, OA increased cell death by about 30%, inducing apoptosis and necrosis in HCC cells but not in healthy hepatocytes at 300 μM dosage. Moreover, OA induced senescence in Hep3B, reduced P-ERK in both HCC cell lines and significantly inhibited the antiapoptotic proteins c-Flip and Bcl-2 in HCC cells but not in healthy hepatocytes. All these results led us to conclude that different cell death processes occur in these two HCC cell lines upon OA treatment. Furthermore, 300 μM OA significantly reduced the migration and invasion of both HCC cell lines, while it has no effects on healthy cells. Finally, we investigated autophagy role in OA-dependent effects by using the autophagy inducer torin-1. Combined OA/torin-1 treatment reduced lipid accumulation and cell death as compared to single OA treatment. We therefore concluded that OA effects in HCC cells lines are, at least, in part dependent on OA-induced autophagy reduction. In conclusion, we report for the first time an autophagy dependent relevant anti-cancer effect of OA in human hepatocellular carcinoma cell lines.

## Introduction

In the last years different research groups investigated the relationships between fatty acids and solid tumors. Fatty acids are major components of biological membranes and play important roles in the intracellular signaling pathways. They are chemically classified as saturated and unsaturated (monounsaturated and polyunsaturated) fatty acids and their structure affects their biological effects. One of the most abundant fatty acid is the monounsaturated fatty acid Oleic Acid (OA), representing the main component of olive oil (70–80%). Olive oil has beneficial effects in counteracting liver steatosis and cardiovascular diseases (Perez-Martinez et al., 2011; Perdomo et al., 2015; Zeng et al., 2020). OA effects on cancer cells are not completely elucidated although they seem to be different depending on cancer cell types (Sales-Campos et al., 2013; Maan et al., 2018). Upon OA administration in *in vitro* set up, lipid droplets (LD) formation occurs within the cells (Rohwedder et al., 2014) and inside these compartments neutral lipids are concentrated with mechanisms still largely unclear (Fujimoto et al., 2006). Most eukaryotic cells can store excess neutral lipids within LD (consisting mainly of triglycerides and cholesteryl esters), and release them when necessary, depending on cellular needs. This property is particularly important in cells exposed to feeding periods followed by starvation periods, such as cancer cells (Jarc and Petan, 2019). In the present study we investigated *in vitro* the effects of OA in HCC models. Previous works have shown that OA treatment leads to a massive lipid accumulation in hepatocytes cell lines (i.e., LO2 and HepG2 cells) associated with cell viability reduction (Yao et al., 2011). We tested whether OA affects lipid accumulation, autophagy and cell death in different HCC cell lines compared to immortalized healthy hepatocytes. Autophagy is a catabolic process essential to maintain cellular homeostasis; it allows the turnover of cellular components including LD (Giampietri et al., 2017). In the autophagy-mediated lipolytic process, LD are associated with the autophagosome protein microtubule-associated protein light chain 3 (LC3) and then are delivered to lysosomes (Singh et al., 2009). Therefore, autophagy plays a crucial role in LD degradation regulating fatty acids mobilization. On the contrary, autophagy impairment, achieved by genetic knockdown of autophagy genes (i.e., atg5 or atg7), significantly increases hepatic lipid stores (Amir and Czaja, 2011). Autophagy is the main cellular response to nutrients deprivation (Denton et al., 2012) and plays a dual role in neoplastic transformations (Mizushima, 2007; D'Arcangelo et al., 2018). Autophagy upregulation under chemotherapy treatment may increase cancer cell survival (Ding et al., 2011). Autophagy inhibition leads to cell death promotion and cell growth inhibition, and its activation induces cell proliferation in HCC (Chava et al., 2017). For such reasons inhibiting the autophagy pathways might be crucial to induce cancer cell death (Tomaipitinca et al., 2019). Relatively little is known about the molecular mechanisms underlying the OA effects in liver cancer cells and the role of autophagy (Li et al., 2014; Maan et al., 2018). Evidences exist showing an inverse relation in liver between levels of autophagy and perilipin-2, a constitutive protein of LD. High levels of Perilipin-2 inhibit LD degradation by decreasing autophagy while perilipin-2 deficiency increases autophagy leading to LD breakdown (Singh et al., 2009; Sanchez-Martinez et al., 2015; Tsai et al., 2017). Further evidences demonstrated a direct relationship between OA and perilipin-2 accumulation in tumors such as glioblastoma, confirming the relationship between OA and LD storage (Taib et al., 2019). Conversely, the role LD store plays on controlling HCC growth is still partially unknown. In the present work we investigated LD accumulation in HCC cell lines (Hep3B and Huh7.5) vs. immortalized healthy hepatocytes (THLE-2) after OA treatment, with a focus on autophagy role. We report an anti-tumor action of OA in HCC and a specific OA effect on lipid accumulation, viability, proliferation, migration and invasion, at least partially dependent on reduced autophagy.

## Materials and Methods

### Cells Culture and Reagents

Hep3B and Huh7.5 cell lines were kindly donated by Professor Maria Rosa Ciriolo “Tor Vergata” University of Rome.

The two HCC cell lines display respectively deletion (i.e., Hep3B) or point p53 mutation (i.e., Huh7.5) as tumor suppressor *p53* is one of the most frequently mutated genes in liver cancer (Rebouissou and Nault, 2020). Cells were cultured in DMEM (Gibco-Invitrogen, Carlsbad, CA, USA) containing high glucose enriched with 10% fetal bovine serum, glutamine (2 mmol/l), in presence of penicillin (100 U/ml) and streptomycin (100 μg/ml). Cells were maintained at 37°C in a humidified 5% CO_2_ atmosphere. OA was purchased from Sigma-Aldrich (Milano, Italy) and diluted with 0.1% NaOH, 10% delipidated BSA (Sigma-Aldrich).

Control cell line (THLE-2) was purchased from the American Type Culture Collection (ATCC, Manasses, VA, USA). THLE-2 cells show phenotypic characteristics of normal adult hepatocytes, are non-tumorigenic when injected into athymic nude mice and do not express alpha-fetoprotein (Pfeifer et al., 1993). THLE-2 were cultured with BEGM Bullet Kit (Catalog No. CC-3170) from Lonza (East Rutherford, NJ, USA). The Bullet Kit contains BEBM Basal Medium (CC-3171 Lonza) and supplements. The final growth medium consists of BEBM supplemented with 10% FCS, bovine pituitary gland extract, hydrocortisone, epidermal growth factor (EGF), insulin, triiodothyronine, transferrin, retinoic acid, 6 ng/ ml human recombinant EGF (Sigma-Aldrich) and 80 ng/ ml o-phosphorylethanolamine (Sigma-Aldrich). THLE-2 cells require a special flask coating medium that consists of the following reagents: a mixture of 0.01 mg/mL fibronectin from human plasma (Sigma-Aldrich), 0.03 mg/mL bovine collagen type I (Sigma-Aldrich) and 0.01 mg/mL bovine serum albumin (Sigma-Aldrich) in BEBM medium.Before seeding, 3 ml of coating medium for a T-75 flask and 1 ml of coating medium for one 6-well plate were applied for 2 min and then aspirated.

ATCC guidelines for culturing THLE-2 are available at: https://www.lgcstandards-atcc.org/products/all/CRL-2706.aspx?geo_country=it#culturemethod.

Hep3B, Huh7.5 and THLE-2 cells were cultured in T-75 flasks and experiments were performed in 6-well plates. The day after plating, cells were treated with OA at different concentration (50, 150, and 300 μM OA) for the indicated time. Bafilomycin A1 was purchased from Sigma-Aldrich and was used at 100 nM during the last 3 h treatment. Torin-1 was purchased from (Tocris, Bristol, UK) and was used during the last 4 h treatment at the concentration of 250 nM for Hep3B and 500 nM for Huh7.5 cell lines.

### Western Blotting

Cells were washed two times with pre-chilled PBS (Phosphate Buffered Saline) purchased from Sigma-Aldrich and lysed. Lysis Buffer 10x (Cell Signaling, Danvers, MA, USA) was diluted in the presence of 2% SDS (Sodium Dodecyl Sulfate) and proteases' inhibitors (Sigma-Aldrich). Lysates were also sonicated through a sonicator (Branson, Danbury, USA) for 10 s at 50% amplitude. Lysates were then incubated for 10 min on ice and then centrifuged at 4°C for 15 min at 14,000 g to remove cell debris.

Protein concentration was determined by micro BCA assay (Pierce, Thermo Scientific, Rockford, IL, USA) and samples were boiled at 95°C for 5 min following Laemmli Buffer addition (0,04% Bromophenol blue, 40% Glycerol, 2% SDS, 20% ß-mercaptoethanol, 250 mM Tris HCl pH.6.8, all purchased from Sigma-Aldrich) (Giampietri et al., 2006).

Proteins were separated by SDS–PAGE and transferred on Polyvinylidene fluoride (PVDF) or Nitrocellulose membranes (Amersham Bioscience, Piscataway, NJ, USA). Membranes were probed using the following antibodies: anti-β-Actin-HRP (Sigma-Aldrich 1:10,000); anti-Tubulin (Sigma-Aldrich 1:10,000); anti-LC3 (Cell Signaling 1:1,000); anti-Perilipin-2 (Sigma-Aldrich 1:500); anti-Cleaved caspase-3 (Cell Signaling 1:700); anti-PARP (Cell Signaling 1:1,000); anti-pERK (Cell Signaling 1:1,000); anti-ERK2 (Santa Cruz, Santa Cruz, CA, USA 1:1,000); anti-Bcl-2 (Santa Cruz 1:500); anti-Flip (Cell Signaling 1:1,000); anti-Cyclin D1 (Santa Cruz 1:500); anti-PCNA (Santa Cruz 1:500); anti-Srebp-1 (Santa Cruz sc-13551 1:50); anti PPAR-gamma (Cell Signaling 2443 1:500).

Secondary antibodies were horseradish peroxidase-conjugated anti-mouse or anti-rabbit (Bio-Rad, Hercules, CA, USA). Membranes were washed with Tris-buffered saline (Medicago, Uppsala, Sweden) with 0.1% Tween-20 (Sigma-Aldrich) and developed through the chemiluminescence system (Amersham Bioscience) on the ChemiDoc image analyser (Bio-Rad, Hercules, CA, USA), Image lab software was used for densitometric quantifications.

### Oil-Red O Staining

Briefly, a stock oil red solution was prepared diluting 0.7 g Oil Red O with 200 mL isopropanol. A working dilution was then obtained by mixing 6 parts Oil-Red O stock with 4 parts dH_2_O. Cells were fixed with 10% formalin 5 min at room temperature. Then fresh formalin was added and incubated 1 h. After formalin removal, cells were washed with 60% isopropanol 5 min at room temperature. After isopropanol removal, oil red working solution was added for 10 min. Cells were then washed with H_2_O and analyzed immediately by light microscopy. The Axioskop 2 plus microscope (Carl Zeiss Microimaging, Inc., Milan, Italy) was used. Images were obtained at room temperature using AxioCamHRC camera (Carl Zeiss Microimaging, Inc.) by Axiovision software (version 3.1, Carl Zeiss Microimaging, Inc.). Then, the stained lipid droplets were dissolved in 1.5 ml 100% isopropanol 5 min at room temperature and the absorbance was measured at 500 nm to quantify neutral lipid accumulation.

### Alamar Blue Assay

Alamar blue assay was performed using Resazurin sodium salt solution (Sigma-Aldrich). Cells were cultured and treated in 96-well plates as previously described, washed and then Resazurin sodium salt solution was added for 4 h. The solution was collected and detected using a luminometer (Promega, Madison, WIS, USA) using 580–640 nm emission filter and 520 nm excitation filter.

### Cell Viability Assay

Cell viability was performed by counting cells in the presence of trypan blue. Cells were seeded on 6-well plates and incubated at 37°C in 5% CO_2_ overnight, then treated with different doses of OA. After OA incubation, cells were detached, volume mixed 1:1 with trypan blue and counted. The percentage of trypan blue positive-dead cells respect to the total cell number was expressed as the viability rate.

### Flow Cytometry Cell Cycle and Cell Death Analysis

For cell cycle analysis, cells were treated with OA at a concentration of 300 μM for 48 h and then the cells were fixed with 70% ethanol, washed three times with PBS and stained for 3 h at room temperature with PBS containing 20 μg/mL RNase A and 50 μg/mL propidium iodide (PI). Around 10,000 cells were analyzed using a CyAn ADP flow cytometer (Beckman Coulter, Brea, CA, USA) and FCS express 5 (*De Novo* software, Glendale, CA, USA). The experiment was performed three times with consistent results.

Annexin Pacific Blue /PI kit (Termo Fisher Scientific, Rockford, IL, USA) was employed for the detection of percentage of cell death according to manufacturer's instructions. Cells were treated with OA at the different concentrations into a 6-well plate at the density of 1 × 10^5^ cells/well for 24 h. Double staining was used to identify the cell membrane phosphatidylserine externalization and PI uptake. The results are from three independent experiments (*n* = 3). Samples were run on the CyAn ADP flow cytometer (Beckman Coulter) and analyzed with FlowJo software, version 10.5.3.

### Wound-Healing Assay

To evaluate cell migration we performed the wound-healing assay using double well culture inserts (Ibidi GmbH, Martinsried, Germany). Each insert was placed in a 24-well plate, 3.5 × 10^4^ cells were plated into both wells of each insert with 70 μL medium containing 10% FBS. When cells were confluent, the culture inserts were gently removed and cells were fed with 1% FBS DMEM (CTRL) or treated with OA 300 μM (in the presence of 1% FBS DMEM). Each well was photographed at 10× magnification immediately after insert removal, for the measurement of the wound (cell-free) area (T0 area considered as 100%), and after 24 and 48 h with a Nikon DS-Fi1 camera (Nikon Corporation, Tokyo, Japan). The mean percentage of residual open area compared with the respective cell-free space taken at T0 was calculated using ImageJ v 1.47 h software. For each experimental condition, three independent experiments were performed.

### Invasion Assay

To determine the invasion ability of HCC cell lines, transwell membrane filters (8 μM pore size) (Falcon, Corning, NY, USA) coated by reduced growth factor matrigel (BD, Franklin Lakes, NJ, USA) were used. 1 × 10^5^ cells were seeded in the upper chamber with 1% FBS medium, 20% FBS medium was added to the bottom chamber. Following 48 h incubation, the cells were removed from the top surface of the membrane. The invasive cells adhering to the bottom surface of the membrane were fixed using 4% paraformaldehyde (Electron Microscopy Sciences, Hatfield, PA, USA) and stained with 600 nM DAPI (Thermo Fisher Scientific, Rockford, IL, USA). The total number of DAPI-stained nuclei of invading cells were counted under a fluorescence microscopy by using ImageJ software in five randomly chosen macroscopic fields per membrane. Each experiment was performed in triplicate and was repeated at least three times.

### β-galactosidase Assay

All the experiments were performed using the beta-galactosidase staining kit according to manufacturer's instructions (Cell Signaling Technologies - USA, Danvers, MA).

Briefly, 100,000 cells were plated on 35 mm Petri dishes at 37°C in 5% CO_2_ overnight, then treated with 300 μM OA for up to 48 h. Cells were fixed at 48 h, then 1 ml of beta-galactosidase staining solution was applied to each dish. Cells were incubated overnight in a dry, CO_2_-free incubator, then were examined under light microscope at 200x magnification. For the quantification of β-galactosidase positive cells, a score from 1 to 3 was assigned to each cell based on color intensity. The average of the scores of three microscopic fields from each Petri dish was calculated and the values were divided by the overall number of analyzed cells. Each experiment was performed in triplicate and was repeated at least three times.

### Statistical Analysis

All the experiments were repeated at least 3 times. Statistical analysis was performed using Prism software (GraphPad). Values are expressed as mean, with individual experiments data points plotting. The statistical significance was determined performing unpaired Student *t*-tests or One-Way Analysis of variance (ANOVA). Student's *t*-test was used for statistical comparison between means where appropriate (two groups) and One-Way ANOVA (three or more groups); *P* ≤ 0.05 was considered statistically significant.

## Results

### Lipid Accumulation Induced by OA Administration

**Figure 1:**
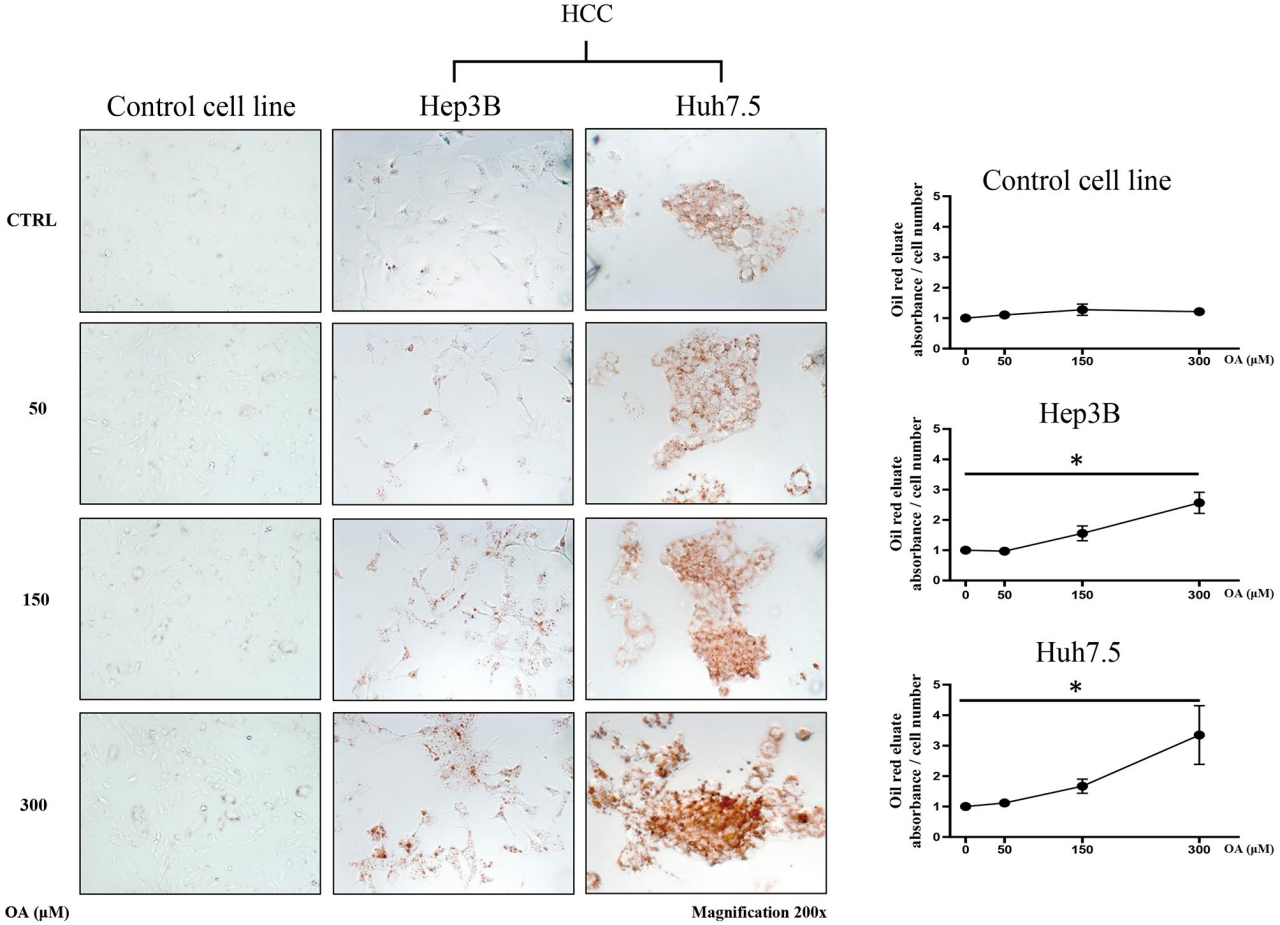
Neutral lipid accumulation upon OA treatment. Control (THLE-2), Hep3B and Huh7.5 cell lines treated with OA. In the left panels: images of the three cell lines: Control, Hep3B and Huh7.5 stained with Oil-Red O after treatment with increasing doses of OA (50, 150, and 300 μM). In the right end panels: quantification of Oil-Red O eluates/cell number, upon treatment with increasing doses of OA (*n* = 3; *p < 0.05).

In order to evaluate the involvement of OA in the modulation of neutral lipid accumulation in human hepatocellular carcinoma and hepatocyte cell lines, we treated Control cell line (THLE-2), Hep3B and Huh7.5 with increasing doses of OA (50, 150, and 300 μM). Upon 24 h treatment, cells were fixed and stained with Oil-Red O dye, which binds neutral lipids, such as triglycerides and cholesterol esters. As shown by optical microscopy analyses, the treatment with increasing doses of OA induced a consistent relevant and dose-dependent Oil-Red O accumulation compared to the basal level into the cytoplasm of both HCC cell lines. Only a slight Oil-Red O staining increase was observed in the control cell line (Figure 1). Oil-Red O quantification by eluate absorbance normalized by cell number, showed a dose dependent increase with a significant value at 300 μM OA vs. untreated cells in HCC.

Supplementary Figure 1 shows a similar increase of oil-red staining at 48 h, suggesting that there is not a delay in lipid accumulation, rather, a permanent increase is present in cancer cells at 24 and 48 h.

### Autophagic Flux and Perilipin-2 Modulation Upon OA Treatment

**Figure 2:**
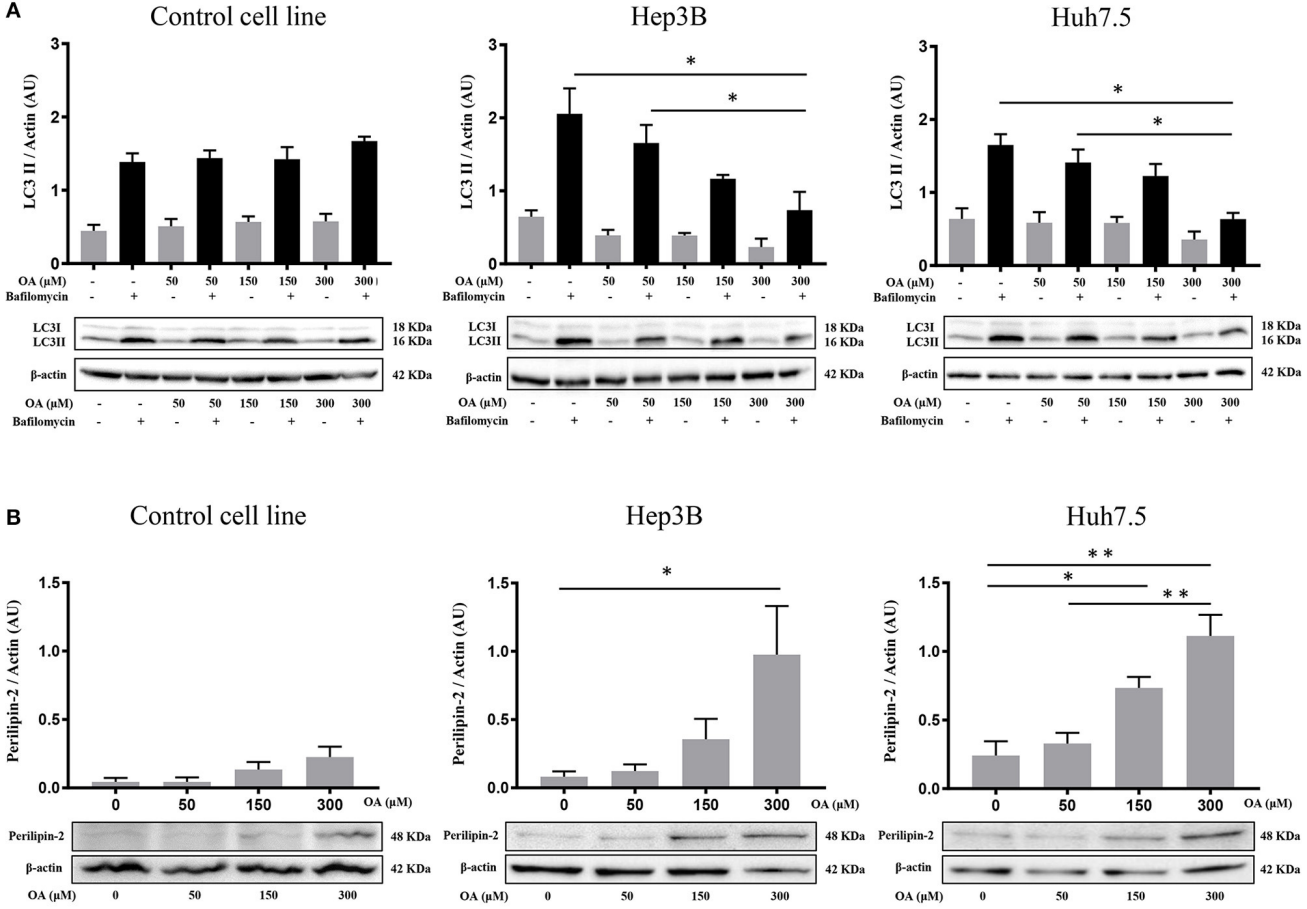
Autophagic flux and perilipin-2 modulation upon OA treatment in Hep3B, Huh7.5 and THLE-2 cell lines. **(A)** Control (THLE-2), Hep3B and Huh7.5 and cell lines treated with OA increasing doses in the presence of bafilomycin A1. LC3II quantification reveals a significant reduction of autophagic flux upon high OA doses in both HCC cell lines, but not in the healthy hepatocyte cell line. **(B)** Western blot analyses for perilipin-2, were performed. Perilipin-2 levels in both HCC cell lines are increased in a dose dependent manner, while in Control cells perilipin-2 levels do not significantly increase upon 48 h OA treatment (*n* = 3; *p < 0.05; **p < 0.01).

Since autophagy is known to be involved in tumor metabolism and in LD break-down, we investigated OA effect on autophagy. We treated Control, Hep3B, Huh7.5 with increasing doses of OA and bafilomycin A1. The presence of bafilomycin A1 allows to evaluate the autophagic flux (Klionsky et al., 2016) by blocking the fusion between autophagosome and lysosome and inducing autophagosomes accumulation. As shown in Figure 2A, increasing doses of OA reduce the autophagic flux in a dose dependent manner, in both HCC cell lines. On the contrary, in the control cell line (THLE-2) OA shows no effect. We speculate that the reduction observed in Figure 2 on Hep3B and Huh7.5 may be associated with the parallel increase observed in Figure 1, while the lack of effect in control cell line is consistent in Figures 1, 2. In order to better understand the relation between LD accumulation and autophagy, the levels of perilipin-2 were investigated by western blot analyses upon 48 h OA administration. Perilipin-2 is located in LD peripheral zone and its abundance is inversely related to autophagy level in liver (Tsai et al., 2017). In Figure 2B perilipin-2 levels in Control, Hep3B, and Huh7.5 cell lines are shown. 48 h OA treatment led to a significant and dose-dependent increase of perilipin-2 levels in both HCC cell lines. Metabolic and inflammation related targets (Zhong et al., 2018; Gnoni et al., 2019) were differently modulated in HCC cells as compared to control cells thus indicating that OA exerts different effects in healthy vs. HCC cells as a consequence of different lipid accumulation (Supplementary Figure 2).

These results show that OA treatment directly affects perilipin-2 expression in hepatocellular carcinoma cell lines, correlating with both neutral lipid accumulation and autophagic flux reduction.

### Viability and Cell Death Upon OA Treatment

**Figure 3:**
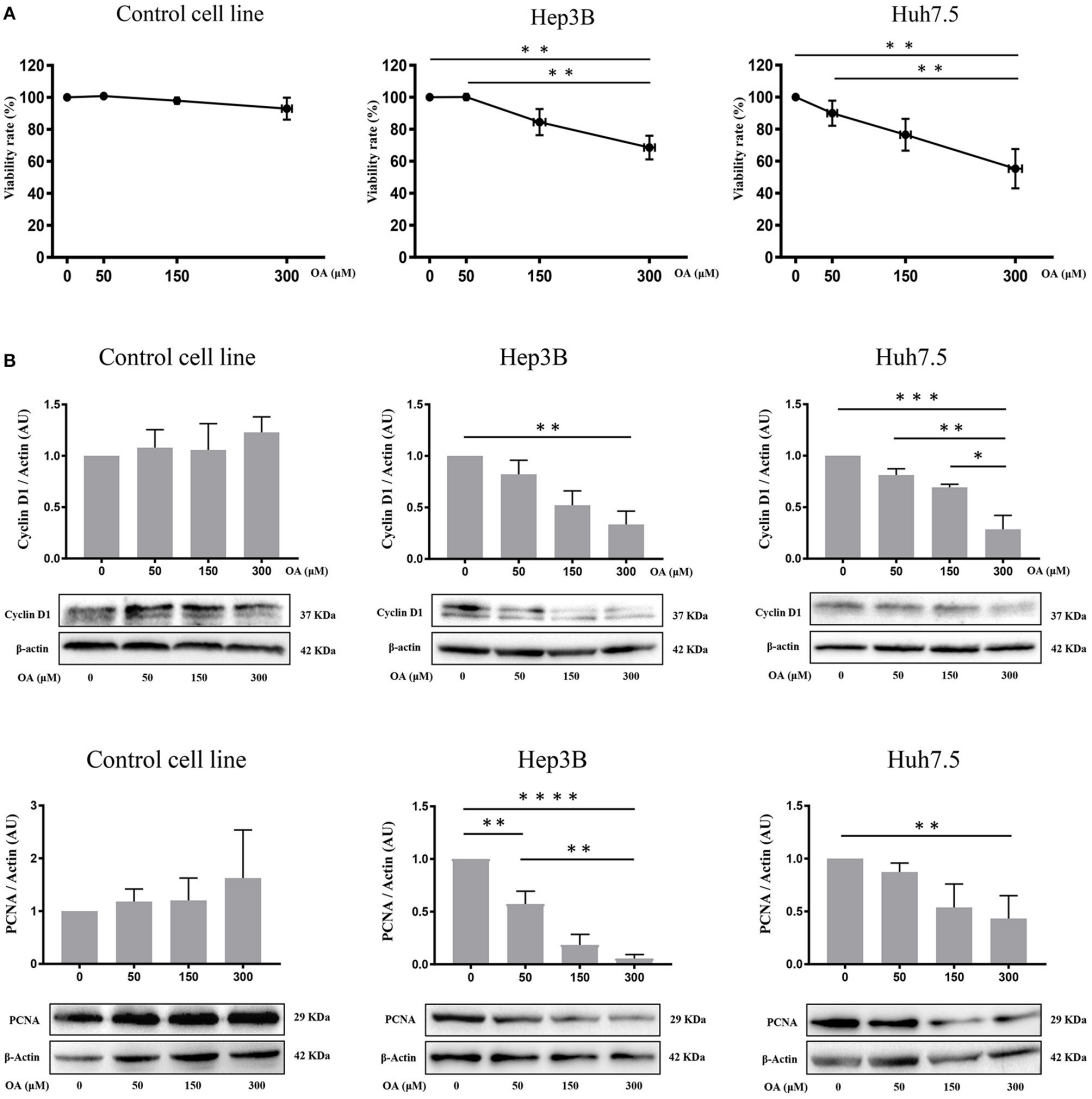
OA reduces viability, cyclin D1 and PCNA in HCC cell lines. Cell viability assays and western blot analyses for Cyclin D1 and PCNA were performed. **(A)** OA induced a significant dose-dependent reduction of cellular viability in HCC cell lines but not in healthy hepatocyte cell line, measured by Alamar Blue assay. **(B)** Western blot Cyclin D1 and PCNA analyses showed that OA treatment induced a significant reduction of Cyclin D1 and PCNA levels in HCC cell lines, but not in healthy hepatocytes cell line (*n* = 3; *p < 0.05; **p < 0.01; ***p < 0.001; ****p < 0.0001).

Control, Hep3B and Huh7.5 cells were treated with OA for 48 h to investigate OA effects on viability and cell death. Alamar Blue assay showed a specific dose-dependent reduction of cellular viability in both HCC cell lines (Figure 3A). Also, OA-dose-dependently reduced the expression of the proliferation markers cyclin D1 and PCNA in both HCC cell lines but not in healthy controls (Figure 3B).

**Figure 4:**
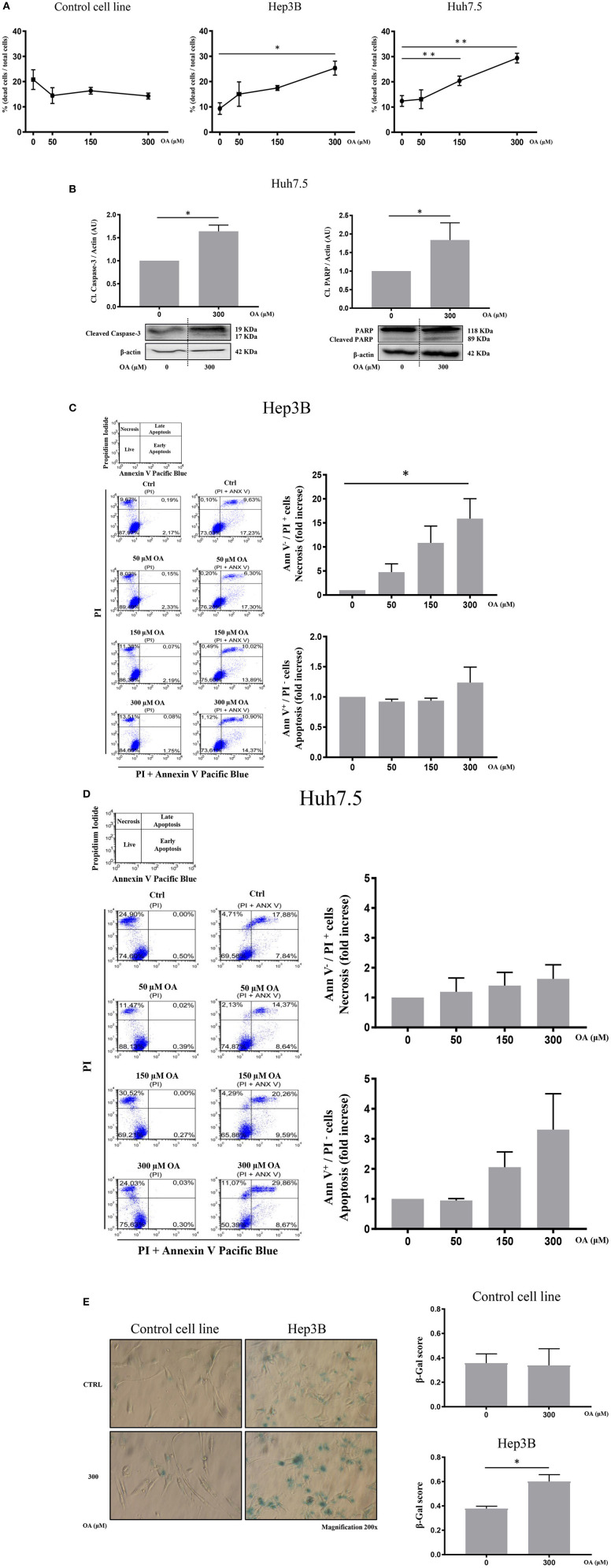
OA increases cell death in HCC cell lines but not in healthy hepatocyte cell line. Dead/total cells percentage ratio, western blot analysis for the cleaved forms of caspase-3 and PARP, cytofluorimetric analysis for Ann V/PI as well as β-galactosidase staining were performed. **(A)** Trypan blue staining showed that OA treatment induced cell death in both HCC cell lines, but not in healthy hepatocytes cell line. **(B)** Western blot analysis for the cleaved forms of caspase-3 and PARP proteins showed that 300 μM OA induced apoptotic cell death in Huh7.5 cell line. **(C,D)** Cytofluorimetric analysis for Ann V/PI staining of Hep3B and Huh7.5 cell lines cultured with different concentration of OA (50, 150, and 300 μM). The strategy of cytometric analysis is showed on the left: representative dot plots from five different experiments, by using PI staining alone for gating Ann V + / PI + cells. On the right, histograms of Ann V – / PI + necrotic cells (fold increase) of Hep3B and Huh7.5 cell lines showed that 300 μM OA significantly induces necrosis in Hep3B (*n* = 3; *p < 0.05; **p < 0.01). **(E)** β-galactosidase staining for Control cell line and Hep3B was performed. Images and graphs revealed that 300 μM OA treatment induced significant increase of senescence phenotype in Hep3B but not in Control (*n* = 3; *p < 0.05).

Then, we evaluated cell death by trypan blue cell staining. Forty-eight hours OA treatment induced a significant cell death in both HCC cell lines, but not in the Control cell line (Figure 4A). Finally, we investigated two markers of the apoptotic pathway, namely, caspase-3 and PARP. Western blot analyses show that both Caspase-3 and PARP are activated by cleavage in Huh7.5 cell line upon 300 μM OA treatment (Figure 4B). Conversely in Hep3B and in Control cells no increase of the active form of Caspase-3 proteins has been observed. Nevertheless, a small sub-G1 population is observed through Flow Cytometry cell cycle analysis after PI staining, suggesting a week apoptotic response in Hep3B (Supplementary Figure 3).

We also carried out cytofluorimetric analyses with Annexin V-FITC/PI. As shown in Figures 4C,D, increasing doses of OA significantly increase necrosis in Hep3B but not in in Huh7.5. Necrosis appears as a dose dependent effect of OA treatment in Hep3B but not in Huh7.5. Finally, as shown in Figure 4E, 300 μM OA treatment significantly induced a senescence phenotype in Hep3B cell line, but not in Control cell line.

We therefore concluded that OA may induce cell death and senescence pathways in HCC cell lines.

### p-ERK and Anti-apoptotic Proteins Modulation Upon OA Treatment

**Figure 5:**
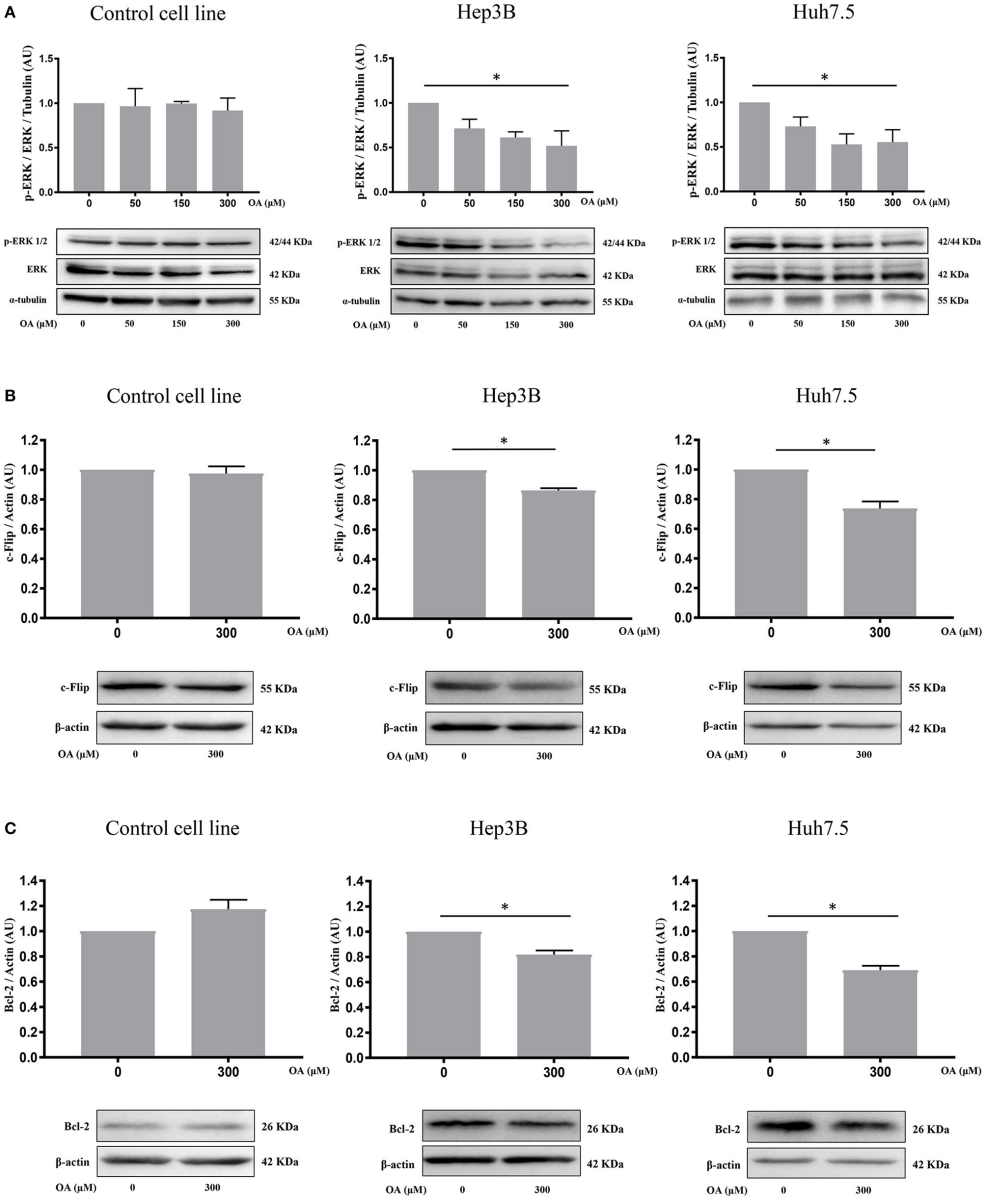
OA significantly reduces ERK phosphorylation and anti-apoptotic proteins in HCC cell lines. **(A)** Western blot analyses show that p-ERK levels are significantly decreased upon 300 μM OA treatment in both HCC cell lines, but not in Control cell line. **(B)** c-Flip levels are significantly reduced in both HCC cell lines, but not in the healthy cells. **(C)** Bcl-2 protein expression is significantly decreased in both HCC cell lines but not in the healthy cells. (*n* = 3; *p < 0.05).

We then investigated p44/p42 MAPK (ERK1/2) phosphorylation after 48 h OA treatment since reduction of ERK phosphorylation in the Thr202/Tyr204 has been related to reduced proliferation and increased cell death (Hennig et al., 2010). Western blot analyses (Figure 5A) show that increasing OA concentrations dose-dependently reduce p-ERK in both HCC cell lines but not in the healthy controls.

To further investigate OA-induced cell death pathways, we treated Control, Hep3B and Huh7.5 cell lines for 48 h with 300 μM OA. Western blot analyses revealed that OA significantly down-regulated the expression of anti-apoptotic proteins c-Flip (Figure 5B) and Bcl-2 (Figure 5C) in both HCC cell lines but not in the heathy cells. These data highlight OA as a possible inducer of cell death processes in HCC by modulating cell death regulators (Tsujimoto et al., 1997; Giampietri et al., 2014). Our results are in agreement and extend previous results obtained in different cellular models showing Bcl-2 reduction upon OA treatment (Jiang et al., 2017).

### OA Reduces Migration and Invasion of Both HCC Cell Lines

**Figure 6:**
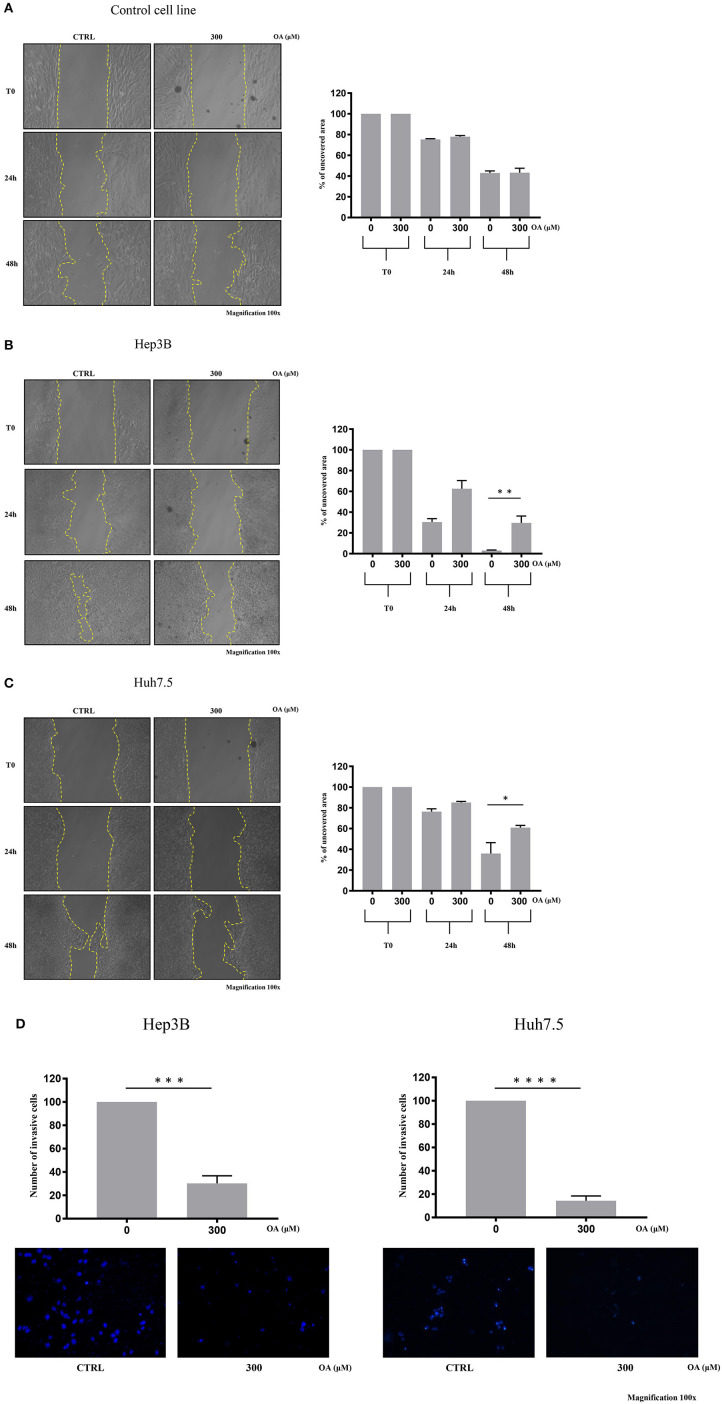
OA reduces migration and invasion of both hepatocarcinoma cell lines. Wound-healing assay on Control **(A)**, Hep3B **(B)**, and Huh7.5 **(C)** cell lines were performed. Representative phase-contrast images wound-healing assay (scratch test) taken at different time points (0, 24, and 48 h) after 300 μM OA treatment are shown. Quantitative analysis of the percentage of uncovered area at 48 h revealed a statistical significance difference in both HCC cell lines after OA treatment, while no differences in Control cell line were observed upon OA treatment (*n* = 3; *p < 0.05; **p < 0.01). **(D)** Invasion assay of Hep3B and Huh7.5 cell lines was performed. Top: significant reduction of invading cells percentage after 48 h OA treatment in both HCC cell lines. Bottom: Representative images of Hep3b and Huh7.5 DAPI-stained nuclei after 300 μM OA treatment are shown (*n* = 3; ***p < 0.001; ****p < 0.0001).

We then performed wound-healing assays to evaluate cell migration. Representative images are shown in Figure 6 at different times after wound scratch. The percentage of uncovered area at different time points represents the different wound recovery ability of Control, Hep3B and Huh7.5 cell lines. Hep3B cells display higher ability to cover the plate as compared to Huh7.5. Such result is in agreement with previous data demonstrating higher Hep3B cell line aggressiveness as compared to other HCC cell lines (Slany et al., 2010; Qiu et al., 2015). OA (300 μM) significantly reduces the migration of both HCC cell lines (Figures 6B,C) as compared to healthy cells (Figure 6A). OA appears to be more potent on Hep3B cells, thus indicating the potential utility of OA in aggressive setup.

We then evaluated the OA effect on invasiveness in transwell invasion assays. As shown in Figure 6D, a significant 70-to-80% reduction of invasion after 300 μM OA treatment is observed in both HCC cell lines.

### The Autophagy Activator Torin-1 Reduces OA-Induced Lipid Accumulation and Cell Death

**Figure 7:**
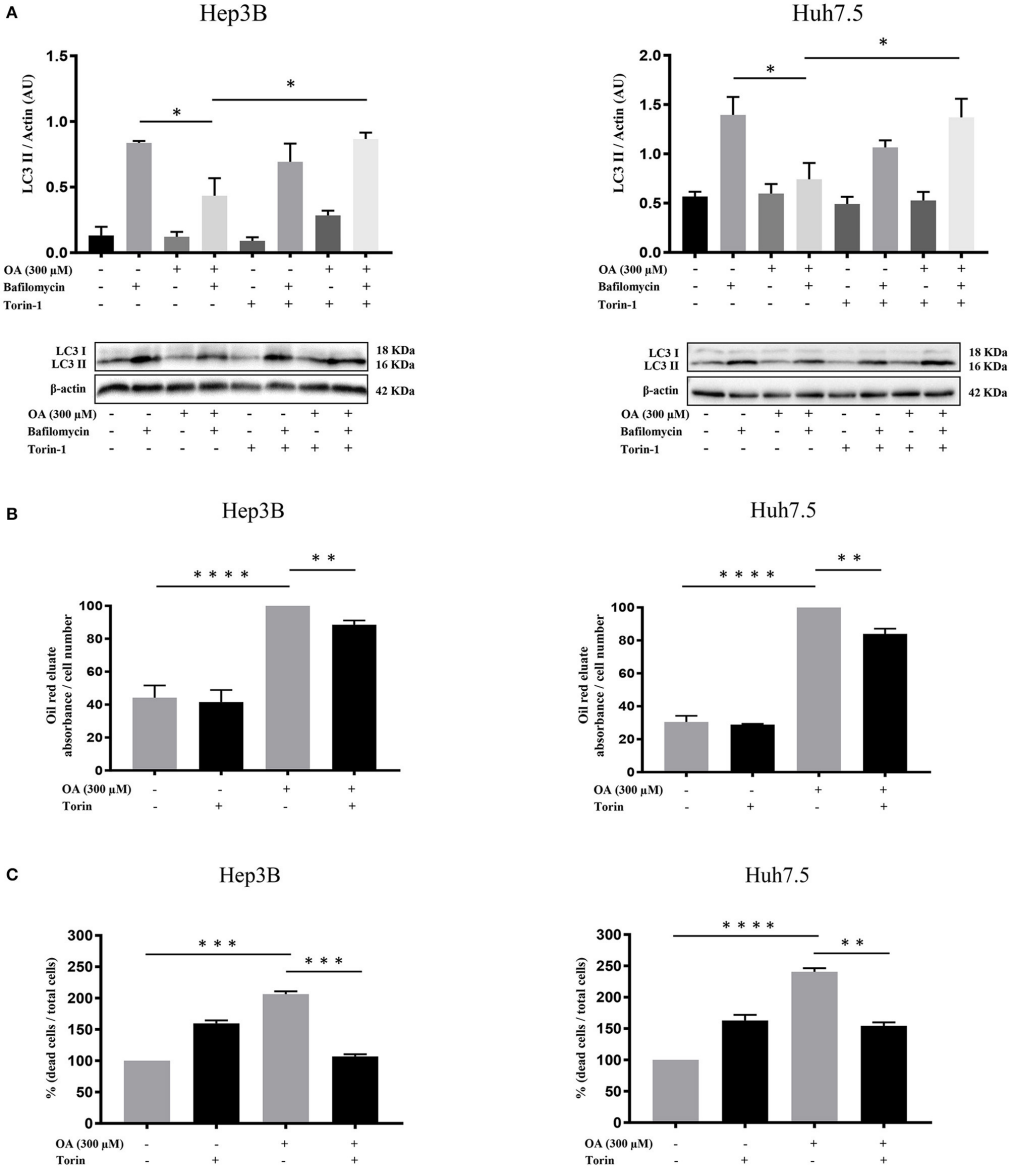
Torin-1 partially reverts the OA-induced effects in HCC cell lines. **(A)** Autophagic flux modulation upon combined OA and torin-1 treatment in both HCC cell lines. Western blot analyses for HCC cell lines treated with 300 μM OA plus torin-1 in the presence of bafilomycin A1 were performed. LC3II quantification confirms that torin-1 activates the autophagic flux after OA treatment in both HCC cell lines. **(B)** Hep3B and Huh7.5 cell lines were treated with OA (300 μM) and torin-1. Oil-Red O-eluates/cell number of Hep3B and Huh7.5 cell lines are shown. OA induced a significant accumulation of neutral lipids in both HCC cell lines; the simultaneous presence of torin-1 significantly decreased the accumulation in both HCC cell lines. **(C)** Cell counts with trypan blue staining were performed. Dead/total cell percentage ratio showed that OA treatment induced cell death in both HCC cell lines; the simultaneous presence of torin-1 significantly reduced the percentage of dead cells in both HCC cell lines (*n* = 3; *p < 0.05; **p < 0.01; ***p < 0.001; ****p < 0.0001).

We then further analyzed the autophagy under OA treatment. Hep3B and Huh7.5 cells were treated with OA combined with torin-1 in the presence of bafilomycin A1. As expected, combined OA/torin-1 treatment increases the autophagic marker LC3II in both HCC cell lines as compared to OA alone (Figure 7A). A significant reduction of neutral lipid accumulation was observed, as compared to single OA treatment in HCC cell lines (Figure 7B). Interestingly, the neutral lipid storage reduction parallels the significant cell death decrease (Figure 7C).

We concluded that OA-induced neutral lipid accumulation and cell death are both dependent on autophagy impairment since the combined OA/torin-1 treatment is able to reduce lipid accumulation and cell death; therefore, OA-dependent anti-tumor effects are dependent, at least in part, on autophagy reduction in HCC cell lines.

## Discussion

In the last recent years different studies highlighted the role of lipids in tumor progression, namely, in hepatocellular carcinoma. This cancer type, like other tumors, exploits lipid reservoirs to promote its progression (Borchers and Pieler, 2010). OA displays important beneficial effects on the liver, by reducing hepatic steatosis and fibrogenesis. OA plays a positive role in the primary prevention of non-alcoholic fatty liver disease (NAFLD). Intake of monounsaturated fatty acids such as OA, may be beneficial for NAFLD patients, as opposed to the intake of carbohydrates, thus reducing the potential risk to develop HCC (Assy et al., 2009). In addition, the effects of OA in different cancer processes are well-known. OA promotes the growth of highly metastatic tumors (Li et al., 2014) while it induces cell death in low metastatic tumors (Carrillo et al., 2012). OA has been also shown to exert anti-cancer effects in tumors inducing lipotoxicity (Yao et al., 2011). In the present study we investigated the involvement of OA in counteracting HCC growth with a particular focus on autophagy. We addressed this issue on two different HCC cell lines vs. healthy hepatocytes. The two HCC cell lines differ in their morphology, growth and cisplatin sensitivity (Qin and Ng, 2002).

Since fatty acids are able to determine LD accumulation in HCC (Jarc and Petan, 2019), we evaluated neutral lipids and LD content. Surprisingly, we observed (Figure 1) a significant increase in neutral lipid storage in both HCC cell lines, but not in the healthy hepatocyte cell line at 300 μM OA, assayed through Oil-Red O staining. We therefore concluded that HCC cell lines display higher attitude to accumulate neutral lipids in LD as compared to healthy cells. As shown in Figure 2 we also found a specific reduction of autophagy marker LC3II and increased LD marker perilipin-2 in HCC thus hypothesizing that autophagy reduction underlies higher LD and neutral lipid accumulation in HCC upon OA administration. An inverse relationship between perilipin-2 and autophagy levels is known to occur in the liver (Tsai et al., 2017) in agreement with our OA-induced effects.

Reduced Alamar Blue staining in both HCC cell lines upon OA treatment as well as significant cyclin-D1 and PCNA decrease in both HCC cell lines (Figure 3) suggest the role of OA as a negative regulator of proliferation in HCC cell lines. Previous studies reported cell proliferation inhibition and apoptosis induction after OA administration in carcinoma cells (Carrillo et al., 2012). In previous works unsaturated fatty acid oleate (an oleic acid-derived salt) induces (Vinciguerra et al., 2009; Park et al., 2018) or inhibits (Arous et al., 2011; Li et al., 2013) HepG2 cell proliferation in a concentration-dependent manner, with a mechanism only partially elucidated. We report here that increasing doses of OA reduce viability and increase cell death (Figure 4) in both HCC cell lines. OA activates the apoptotic process in Huh7.5 but not in Hep3B and increases necrotic cell percentage in Hep3B but not in Huh7.5. Such data agree with previous observations indicating that OA, among many beneficial functions, can induce cell death through apoptotic (Jiang et al., 2017) and non-apoptotic pathways (Yamakami et al., 2014). Remarkably, as described by Magtanong et al. (2016) there are several non-apoptotic cell death pathways activated by OA, such as necroptosis. OA is known to modulate cell death by altering lipid metabolism or by altering membrane lipid composition (Fontana et al., 2013; Ning et al., 2019).

Recently, Bosc et al. (2020) demonstrated that autophagy regulates fatty acids availability through mitochondria-endoplasmic reticulum contact sites and this event occurs mainly in cancer cells. The metastatic potential of cancer cells is related to genes involved in fatty acids synthesis and intracellular lipids storage. Therefore, modulation of lipid accumulation, function of enzymes dedicated to LD digestion, and fatty acids availability play together a role in tumor progression (Sanchez-Martinez et al., 2015; Giampietri et al., 2020). In fact, lipid metabolism generates a high energy support used by cancer cells to grow and metastasize. It is important to note that OA accumulates inside the cell as triglycerides and cholesterol esters, resulting in LD formation, i.e., cellular organelles important in lipotoxicity control (Wen et al., 2013; Petan et al., 2018). Interfering with LD accumulation leads to cell death in fibroblasts exposed to the otherwise non-toxic oleate (Listenberger et al., 2003).

We report here a senescent phenotype in β-galactosidase stained Hep3B after OA treatment (Figure 4). This result is in accordance with our data showing that Hep3B cell line does not undergo apoptosis but necrosis after OA treatment. Different factors are known to regulate cellular senescence and cells displaying G1 or G2 phase increase with S-phase reduction may enter a senescent state becoming resistant to apoptotic signals and undergoing necrosis (Kastan and Bartek, 2004; Gire and Dulic, 2015). Furthermore, senescence observed on OA-treated Hep3B is in accordance with previous reports demonstrating OA as a mild senescence inducer (Iwasa et al., 2003; Yamakami et al., 2014). Further studies are underway to further evaluate cell death processes induced by OA in HCC cell lines. We therefore concluded that in our experimental setup OA activates both apoptotic (Jiang et al., 2017) and non-apoptotic pathways (Assy et al., 2009), depending on cell type.

We observed that OA treatment displays significant reduction of c-Flip and Bcl-2 in both HCC cell lines but not in the healthy hepatocyte cell line (Figure 5). Wang et al. described the anti-apoptotic role of both these proteins in the liver (Wang, 2015). It is well-known that c-Flip has multiple roles, modulating apoptosis, autophagy and necrosis (Safa, 2013). Its up-regulation was correlated with a poor clinical outcome in many pathological conditions including cancer. Moreover, agents or molecules able to inhibit c-Flip expression are of potential therapeutic interest (Safa, 2013). Bcl-2 is known for its properties in cell death modulation and OA has been shown to reduce Bcl-2 expression levels in tongue squamous carcinoma cells (Jiang et al., 2017). In accordance with our results showing OA-dependent cyclin D1 decrease and cell death activation, we also found dose-dependent OA p-ERK reduction, reported in Figure 5. This finding parallels results obtained in tongue squamous cell carcinoma cells, where dose-dependent OA treatment reduced p-ERK1/2 (Jiang et al., 2017). In the present study OA significantly reduced the migratory capability of HCC cells as compared to Control cells (THLE-2) and reduced the number of invading cells in both HCC cell lines (Figure 6). Hep3B cells display higher ability to cover the scratch respect to Huh7.5 cells. This difference between the two cell lines is in agreement with previous data indicating higher aggressiveness of Hep3B cell line vs. other HCC cell lines. Taken together these data supported the hypothesis that OA, by negatively modulating the autophagic flux, counteracts the aggressiveness and invasiveness of Hep3B and Huh7.5 cell lines.

OA treatment in hepatic cell lines like HepG2 or immortalized hepatocytes induces lipid accumulation and represents an *in vitro* model of liver disease (Lim et al., 2020). Under our experimental conditions, OA treatment induces lipid accumulation as expected in healthy cells (THLE-2), although at a lower extent as compared to cancer cells (Hep3B and Huh7.5). This suggests a beneficial role of OA in cancers cells since the higher lipid accumulation observed in cancer cells leads to cell death and to reduced proliferation, migration, and invasion. We demonstrate that this is likely related to autophagy flux reduction in cancer cells. These results agree with Li et al. (2013), who demonstrated that reduced invasiveness of HCC cells (HepG2 and BEL7402) is related to a negative modulation of autophagy. To verify the role of autophagy in OA-dependent effects, HCC cells were treated with OA and analyzed for LD content in the presence of the autophagy inducer Torin-1 (a mTOR kinase inhibitor). The results shown in Figure 7 led us to conclude that 300 μM OA-induced LD accumulation and cell death are both, at least partially, dependent on autophagy impairment since the combined torin-1/OA treatment reduces LD and cell death. Previous studies demonstrated that OA treatment reduces autophagy in Hepa1c1c7 mouse hepatoma cell line (Ning et al., 2019); also the saturated palmitic acid (PA) impairs autophagic-flux in a time-dependent manner in liver HepG2 cells (Korovila et al., 2020). Furthermore, OA was previously shown to exert different effects in HepG2 cells at different concentrations (Pang et al., 2018). In particular LD accumulation and apoptosis induction was reported at concentrations ranging from 0.1 to 2 mM OA while LD reduction was found at 400 μM OA treatment. The Authors concluded that these concentration-dependent effects are strictly related to autophagy since autophagy is able to prevent 400 μM OA-induced HepG2 apoptosis.

Results of the present study achieved on three human cell lines-based *in vitro* systems, confirm the pivotal role of autophagy reduction in promoting OA-dependent LD accumulation, cell death and reduced aggressiveness/invasiveness. Additional studies are needed to further clarify the underlying molecular mechanisms. We conclude that OA stimulates HCC cell death via autophagy reduction while it does not impair autophagy level in healthy cells thus leading us to hypothesize that fine autophagy regulation preserves healthy hepatocytes resistance to toxicity caused by high levels of neutral lipids. LD accumulation in association with autophagic flux reduction after OA treatments in Hep3B and Huh7.5 cell lines, promote cell death through apoptosis in Huh7.5 and also non-apoptotic pathway in Hep3B cell line. Such differences in cell death mechanisms are currently under further investigation.

In conclusion, we present here several evidences indicating OA specific antitumor effects in HCC in an autophagy-dependent manner.

## Data Availability Statement

The raw data supporting the conclusions of this article will be made available by the authors, without undue reservation.

## Author Contributions

FG, SP, and CG conceived the study. FG performed the majority of the experiments and analyzed the data. SM and AD'A performed flow cytometry analyses. LT and VF supported FG in performing some experiments. FG and CG wrote the manuscript supervised by AF, EG, and EZ. All authors contributed to the article and approved the submitted version.

## Conflict of Interest

The authors declare that the research was conducted in the absence of any commercial or financial relationships that could be construed as a potential conflict of interest.
