# Supplementary figures and images for: Anti-tumor Effect of Oleic Acid in Hepatocellular Carcinoma Cell Lines via Autophagy Reduction
Source: Front Cell Dev Biol. 2021 Feb 5;9:629182. doi: 10.3389/fcell.2021.629182 (PMC7892977; doi:10.3389/fcell.2021.629182)

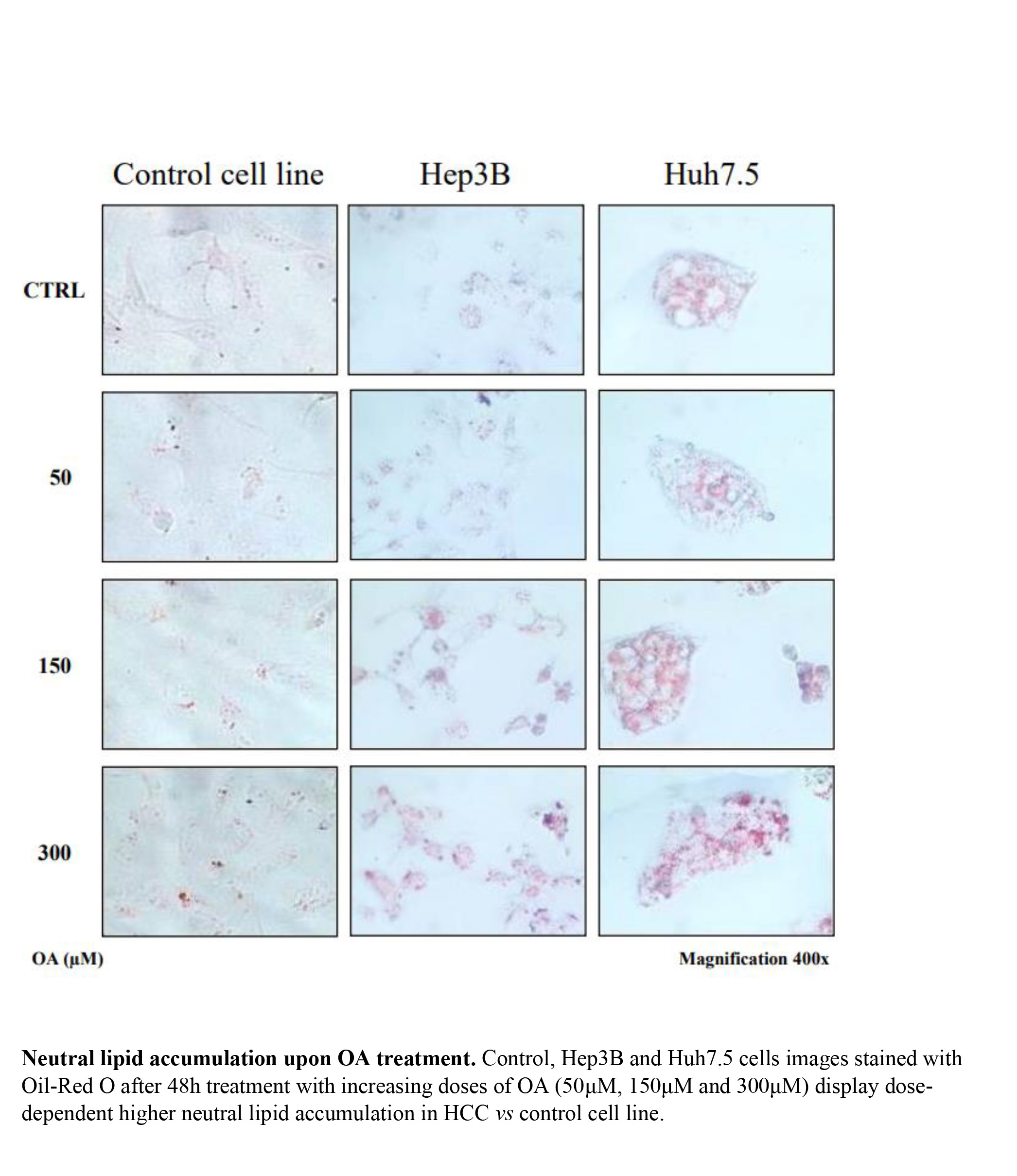

Supplement: Supplementary file 1 [file Image_1.jpg]

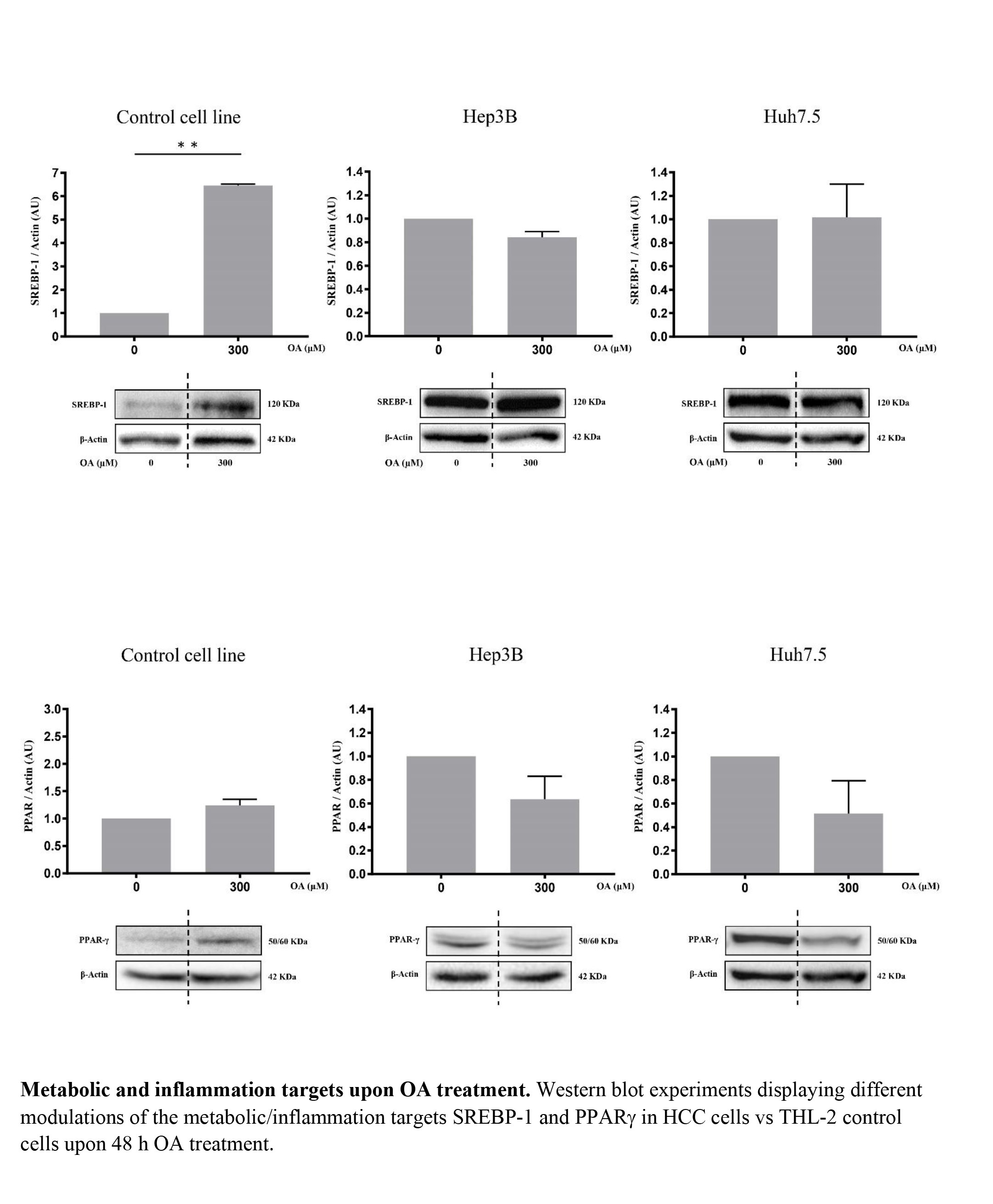

Supplement: Supplementary file 2 [file Image_2.jpg]

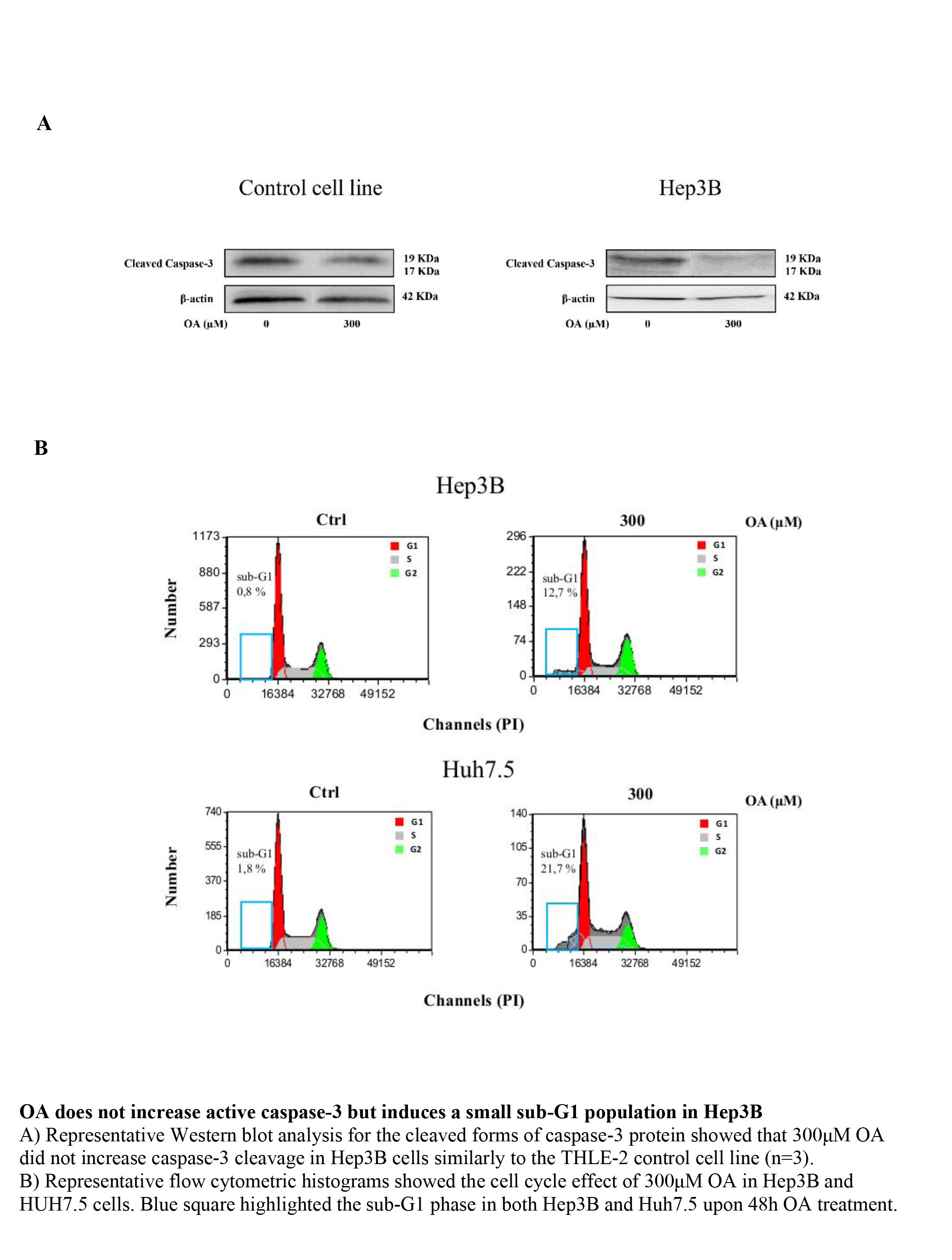

Supplement: Supplementary file 3 [file Image_3.jpg]
